# Incentivierung von Prosumern auf einem elektronischen Marktplatz für personennahe Dienstleistungen

**DOI:** 10.1365/s40702-020-00631-w

**Published:** 2020-07-01

**Authors:** Jan Hofmann

**Affiliations:** Institute of Information Systems, Lehrstuhl für Wirtschaftsinformatik, insbes. im Dienstleistungsbereich, Friedrich-Alexander-Universität Erlangen-Nürnberg, Lange Gasse 20, 90403 Nürnberg, Deutschland

**Keywords:** Motivation, Dienstleistung, Anreizsetzung, Prosument, Motivation, Services, Incentivisation, Prosumer

## Abstract

Im Kontext des BMBF-geförderten Verbundprojekts INSELpro bieten Prosumer ihre Dienste auf einem Markt der örtlichen Nachbarschaftsdienstleistungen an. Hilfebedürftigen Mitbürgern in einem geografischen Quartierbereich werden über die Dienstleistungsplattform zu ihrer jeweiligen Unterstützungsanforderung passende Helfer vermittelt, die sich grundsätzlich dazu bereit erklärt haben, Hilfe zu leisten. Nachfrage und Angebot werden über eine Sharing-Plattform vermittelt, aber außerhalb dieser endgültig vereinbart und erbracht. Die Besonderheit ist dabei, dass die Auswahl der Hilfswilligen für die Anfrager auf Basis einer Matrix aus benötigten und vorhandenen Fähigkeiten eingeschränkt wird, was die Vermittlungsqualität verbessert.

Das Forschungsprojekt setzt auf den wissenschaftlichen Ansatz des Prototyping, bei dem ein Artefakt (hier: die Vermittlungsplattform mit einer App als Frontend) in mehreren iterativen Zyklen entwickelt wird. Dabei sind jeweils nutzerzentrierte Evaluationen direkt an die einzelnen Entwicklungsschritte angekoppelt, um ggf. Fehlentwicklungen direkt gegensteuern zu können.

Um die Beteiligung an dieser elektronisch unterstützten und koordinierten Art der Nachbarschaftshilfe auszubauen bzw. auf einem hohen Niveau zu halten, sind unterschiedliche Maßnahmen denkbar. Der vorliegende Beitrag zeigt Ansätze zur Incentivierung der beteiligten Prosumenten auf, die sowohl online als auch offline durchführbar sind.

## Abstract (en)

In the context of the joint project INSELpro funded by BMBF, prosumers offer their services on a market of local neighbourhood services. Citizens in need of help in the local geographic sector are placed with suitable helpers for their respective support requirements via the service platform, who have basically agreed to provide assistance. Demand and supply are mediated via a sharing platform, but final agreements as well services are provided offline. The special feature here is that the selection of those willing to help the inquirers is limited based on a matrix of required and existing skills, which will improve the quality of the placement.

The research project is based on the scientific approach of prototyping, in which an artifact (here: the mediation platform with an app as front-end) is developed in several iterative cycles. User-centered evaluations are directly linked to the individual development steps to be able to directly counteract any undesirable developments.

Various measures are conceivable to expand participation in this electronically supported and coordinated form of neighbourhood assistance or to maintain it at a high level. This paper describes approaches to incentivising the prosumers involved, which can be carried out both online and offline.

## Einführung

Immer mehr Menschen müssen aufgrund ihrer Lebenssituation bei kurz- oder langfristig auftretenden Be- und Überlastungen Hilfe von Dritten in Anspruch nehmen. Personennahe Dienstleistungen sind immer dann gefragt, wenn Hilfe in persönlichen Bereichen benötigt wird, zum Beispiel bei der Betreuung der Kinder oder im Krankheitsfall. Aber auch alltägliche Verrichtungen wie das Einkaufen, Behördengänge oder hauswirtschaftliche Tätigkeiten können in Verbindung mit den übrigen Beanspruchungen der Menschen zu Überlastungen führen. Häufig bleibt dabei wenig Spielraum für Freizeitaktivitäten, Entspannung und Erholung. Wenn dann noch kurzfristig auftretende Ereignisse die bestehende Tagesplanung zunichtemachen, entsteht Stress, Hektik und Unsicherheit und die Leistungsfähigkeit der Betroffenen sowie ihres sozialen Umfeldes kann leicht an ihre Grenzen stoßen. In ländlichen Regionen klingelt man beim Nachbarn, wenn man kurzfristig und zeitnah Hilfe benötigt. In urbanen Gebieten kennt man seine Nachbarn häufig nicht mehr. Unter diesen Voraussetzungen im Bedarfsfall die Hemmschwelle zu überwinden und mehr oder weniger unbekannte Personen um Hilfe in persönlichen Lebensbereichen zu bitten, ist – speziell für ältere, behinderte oder neu zugezogene Mitbürger – oft sehr schwierig. Bei anderen Bevölkerungsgruppen wie Alleinerziehenden, jungen Familien oder beruflich stark eingebundenen Arbeitnehmern ist festzustellen, dass sie bestehende Dienstleistungsangebote gerne in Anspruch nehmen, wenn ihre finanziellen Möglichkeiten dies zulassen. Das Potential einer gegenseitigen unentgeltlichen Nachbarschaftshilfe bleibt in diesem Kontext jedoch weitgehend ungenutzt.

Langfristig planbare Unterstützungsbedarfe wie regelmäßige Kinderbetreuung (z. B. Kindergarten) bei Alleinerziehenden oder Unterstützungsleistungen durch Pflegedienste (z. B. Körperpflege, Fahrdienste etc.) bei informell Pflegenden und Menschen mit Behinderung können aktuell bereits zuverlässig durch Leistungen sozialer Einrichtungen (z. B. Diakonie) abgedeckt werden (bspw. Zentraler Diakonieverein o.J.; Sozialzentrum der Diakonie o.J.). Kurzfristig oder sehr spontan auftretende Hilfebedarfe sind jedoch auf diesem Wege kaum abzudecken. Oftmals stellt der Rückgriff auf ein Netzwerk von Verwandten und Freunden – soweit ein solches vorhanden ist – aktuell die einzige mögliche Unterstützungsquelle dar. Eine besondere Herausforderung besteht insbesondere dann, wenn diese Helfer beim kurzfristigen Hilfebedarf nicht spontan verfügbar sind. Dies wird im besonderen Maße infolge der administrativen Einschränkungen des öffentlichen Lebens im Zuge der weltweiten Verbreitung des Corona-Virus seit dem Frühjahr 2020 sichtbar.

Die Motivation für das BMBF-geförderte Projekt INSELpro^1^ ist die Sicherstellung der beruflichen und gesellschaftlichen Teilhabe der Betroffenen unter Berücksichtigung kurzfristiger Ereignisse. Das Vorhaben adressiert insbesondere den Einsatz von Informations- und Kommunikationstechnologien zur effizienten Gestaltung und Umsetzung personennaher Dienstleistungen und die Frage, wie sie eine moderierende Rolle zwischen allen Beteiligten einnehmen kann.

Im Projektkontext bieten Prosumer ihre Dienste auf einem Markt der örtlichen Nachbarschaftsdienstleistungen als freies Gut an, das durch die Nutznießer normalerweise nicht entlohnt wird (vgl. Güttel 2007). Der Preis für ein Angebot auf dem Dienstleistungsmarkt der INSELpro-Plattform ist Null. Die Motivation zur Nachbarschaftshilfe muss daher intern vorhanden sein oder von außen angereizt werden (vgl. Rimser 2014). Wichtiger Bestandteil dafür sind organisatorische bzw. umfeldliche Voraussetzungen, wie beispielsweise die Eingrenzung des Aktivitätsbereiches auf den Bereich um und in einem neu entstehenden Stadtquartier^2^ oder die Existenz eines *Quartiersmanagers*, dessen originäre Aufgaben (Betreuung des Service-Wohnens im Quartier) dahingehend erweitert werden, dass auch die inhaltliche und Nutzerverwaltung der Plattform, Hilfestellung zur App oder Werbemaßnahmen vor Ort koordiniert und verantwortet werden. Aber auch, wenn die genannten Voraussetzungen gegeben sind, erscheinen Incentivierungsmaßnahmen sinnvoll (vgl. Bendt 1999). Ihr Hauptzweck ist dabei, die Freude an der Unterstützung hilfebedürftiger Mitmenschen zu stimulieren und so den Austausch auf dem Dienstleistungsmarkt und damit die Nachbarschaftshilfe aktiv zu halten. Dabei fungiert eine IT-gestützte Sharing-Plattform als Marktplatz der örtlich begrenzten Nachbarschaftshilfe: Nachfrager formulieren mittels einer Smartphone-App eine Suchanfrage, die genauer spezifiziert wird durch den Zeitraum der benötigten Unterstützung sowie die Zuordnung zu vordefinierten Hilfekategorien. Ein Algorithmus ordnet im Hintergrund die benötigten Charakteristika der Suche den bei der Registrierung als Nutzer eingestellten und später jederzeit anpassbaren Fähigkeiten und freien Zeitslots potenzieller Unterstützer zu und gibt eine vorgefilterte Liste an Personen aus, welche die zur Hilfestellung geforderten Eigenschaften aufweisen. Hieraus wählt der Suchende die notwendige Anzahl an Unterstützern aus und kontaktiert sie initial über die App. Bei Hilfezusage durch die Angefragten findet die weitere Abstimmung außerhalb der App statt, z. B. über Telefonate oder persönliche Treffen.

Diese Filterung des Ergebnisraumes von Matches auf die Anfrage ist einer der wichtigsten Unterschiede zu bestehenden Netzwerken der Nachbarschaftshilfe, die entweder an sich nur einen spezialisierten Themenkreis adressieren oder eher als digitalisiertes schwarzes Brett zu verstehen sind, dessen Einträge ohne besondere Filterung allen Mitgliedern angezeigt werden. Somit besteht für die potenziellen Unterstützer die Gefahr, für sie relevante Hilfsgesuche zu übersehen.

Letztlich fördert die Nutzung der Plattform den Zusammenhalt und die Lebensqualität im Stadtteil und hilft gleichzeitig dabei, die (durch die zusätzlichen Aufgaben in der Verwaltung der Plattform gestiegene) Arbeitsbelastung des Quartiersmanagers zu senken: Helfen sich die Anwohner durch Vermittlung über die Plattform gegenseitig, so wird nicht der Quartiersmanager als Ansprechstelle für Hilfegesuche belastet.

Die wissenschaftlichen Überlegungen zur Sicherstellung der Prosumer-Motivation im Projekt INSELpro basieren auf Arbeiten aus verschiedenen (Teil‑)Disziplinen, insbes. Wirtschafts- und Sozialpsychologie sowie Wissensmanagement und -transfer. Diese werden in den Kontext personennaher Dienstleistungen transferiert, adaptiert und im Folgenden vorgestellt.

## Basisüberlegungen zur Motivation bei der elektronischen Anbahnung personennaher Dienstleistungen

Barbuto (2005) identifiziert fünf Motivationsquellen. Als intrinsisch, also aus eigenem Antrieb heraus, werden sowohl die Prozessmotivation als auch das interne Selbstverständnis bezeichnet. Erstere wird als das Durchführen einer Arbeit um ihrer selbst willen charakterisiert: Die Aufgabe „macht einfach Spaß“. Als Selbstverständnis ist eine innere Leitlinie zu verstehen, die verfolgt werden soll: Man „kann sich nicht mehr im Spiegel ansehen“, wenn nicht entsprechend gehandelt wird. Als extrinsische Faktoren gelten die instrumentelle Motivation (die Aussicht auf Belohnung), die Internalisierung von Zielen des Unternehmens (man möchte einen Beitrag zum Unternehmenserfolg leisten) sowie das externe Selbstverständnis, welches der eigenen Rolle und den Erwartungen des Umfeldes über die eigenen Arbeitsergebnisse entstammt.

Konkrete Incentivierungsmechanismen von bestehenden Plattformen wurden eruiert und ausgewertet. Ein verbreiteter Ansatz ist die Motivation mithilfe von Badges (Li et al. 2012) als Element der Gamification, in deren Rahmen spieltypische Elemente in spielfremden Kontext zur Steigerung der Motivation und Generierung von User Experience übertragen werden (Deterding et al. 2011). Untersuchungen zeigen, dass Badge-Systeme einen positiven Einfluss auf die Motivation und das Verhalten von Akteuren in Communities haben können (Kusmierczyk und Gomez-Rodriguez 2018). Eine Untersuchung des Einflusses von Badges auf die Beteiligung auf der Online-Hilfeplattform StackExchange (Meng et al. 2013) wurde mit Fokus auf projektrelevante Gruppen mit insgesamt ca. 350.000 Nutzern (u. a. im Bereich Elternhilfe und Heimwerker) durchgeführt und über einen längeren Zeitraum fortgeschrieben. Die Analyse zeigt deutlich den Übergang von registrierten „Free-Riders“ (Nutzer, die nicht selber als Leistungserbringer aktiv werden, sondern nur Dienstleistungen und Hilfen „konsumieren“) hin zu einer aktiven Teilnahme im zeitlichen Zusammenhang mit der Einführung neuer Badges.

Das im Rahmen des INSELpro-Ansatzes über die Plattform entwickelte Incentivierungsmodul wird mit Daten der Angebots‑, Vermittlungs- und Konsumfunktionen versorgt: Dienstleistungsanbieter erarbeiten sich „Fleißsterne“ für eine bestimmte Anzahl unternommener Hilfeleistungen. „Experten“ eines bestimmten Fachgebietes werden über ihre in den Angebotspool eingespeisten Hilfsleistungen, ihre angegebenen Fähigkeiten und ihre Bewertungen identifiziert. Bei erfolgreichem Abschluss der Hilfeleistung steigen sie in der Hierarchie der Prosumer weiter auf. Entsprechende Auszeichnungen werden im Profil des Nutzers aufgeführt und sorgen so auf der Plattform für (virtuelle) Reputation.

Analog dazu kann die Mitarbeit bei INSELpro auch außerhalb der Plattform belohnt werden, wie beispielsweise durch Gratifikationen sowohl monetärer als auch nichtmonetärer Art. Sobald sich ausreichend Prosumenten am Dienstleistungsaustausch beteiligen, sind externe Motivatoren oftmals eher hinderlich (vgl. Aronson et al. 2012), sodass sie lediglich bis zum Erreichen einer gewissen, sich selbst tragenden „Community“ einzusetzen sind. Andernfalls droht ein Verdrängungs- oder Korrumpierungseffekt, der intrinsische durch extrinsische Motivation ersetzt (vgl. u. a. Lepper et al. 1973; Frey und Bohnet 1994 sowie Deci et al. 1999). Dieser ist durch die dem konkreten Projekt innewohnenden „Free-to-use“-Policy vernachlässigbar: Das INSELpro-Motivationskonzept setzt primär auf Plattform-interne Incentivierungsmaßnahmen.

Anforderungen an die Ausgestaltung des Anreizsystems werden in Abschn. 3 im Detail erläutert.

**Abb. 1:**
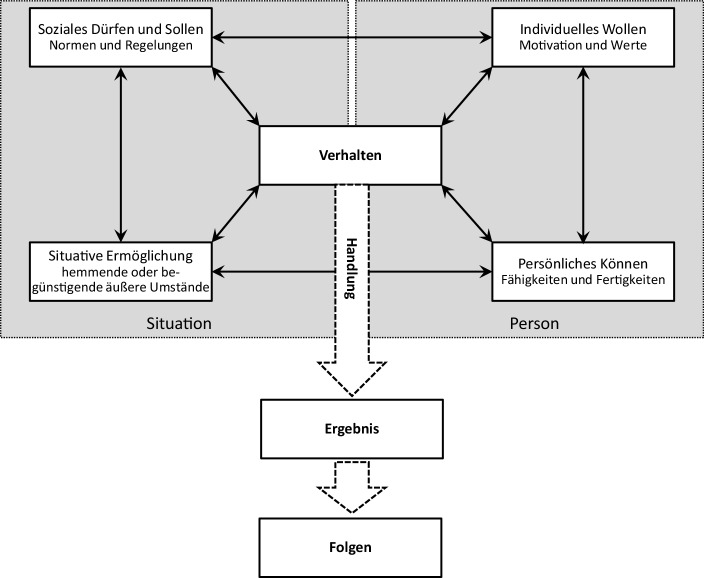
Determinanten und Verlauf menschlichen Handelns. (Quelle: erweitert nach von Rosenstiel 1999 sowie Heckhausen und Heckhausen 2010)

Menschliche Handlungen sind nach von Rosenstiel (1999) durch die *Person* sowie die *Situation* determiniert, in der sich die betrachtete Person befindet. Sie weist spezifische externe Aspekte auf, die sich in Normen und Regelungen (soziale Vorgaben) sowie Umstände einteilen lassen. Auf personeller Seite tragen die Fähigkeiten und Fertigkeiten (das persönliche Können) sowie die eigene Motivationslage und Werte (das individuelle Wollen) zum Verhalten bei. Durch die vielfachen Wechselwirkungen zwischen den genannten Bestandteilen bildet sich das Verhalten, das zu einer Handlung mit Ergebnis und entsprechenden Folgen führt (siehe Abb. 1).

**Abb. 2:**
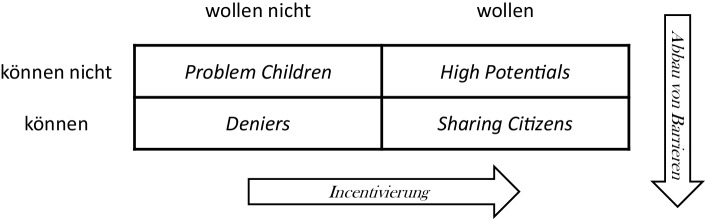
Bereitschaft und Befähigung zur Beteiligung als Prosument. (Quelle: erweitert nach Boeglin 1992, S. 87 sowie Lehner 2014, S. 92 f)

Das menschliche Verhalten kann durch Anreizsysteme beeinflusst werden. Diese umfassen im Projektkontext die vom Plattformbetreiber gezielt dafür eingesetzten Umgebungsbedingungen, die Verhaltensweisen von Prosumern mithilfe positiver Anreize zu fördern oder durch negative Anreize zu reduzieren (vgl. Bea und Haas 2016). Personen lassen sich dabei in Anlehnung an Boeglin (1992) sowie Lehner (2014) in einer Matrix nach „Können“ und „Wollen“ einteilen, die in Abb. 2 dargestellt ist:

1. *Problem Children* können und wollen nicht als Dienstleister in Aktion treten.
2. *Deniers* sind in der Lage, die Plattform zu benutzen (können), sind aber aus unterschiedlichsten Gründen nicht bereit, dies zu tun (wollen nicht).
3. Als *High Potentials* werden diejenigen Plattformbenutzer bezeichnet, die zum Dienstleistungsaustausch motiviert sind (wollen), aber trotzdem nicht aktiv partizipieren (können nicht).
4. Im Gegensatz dazu sind *Sharing Citizens* in der Lage und willens, auf dem Angebotsmarkt der Plattform mitzuwirken. Sie verfügen zumeist über größeres Wissen und besondere Expertise und haben sowohl die Zeit als auch die Kompetenz, mit Dienstleistungen zu unterstützen. Sie sind selbstbewusst, kennen ihren Stellenwert in der Gesellschaft und können den Einfluss ihres Beitrages auf die Community abschätzen.

Es kommt also darauf an, die Gruppen 1 bis 3 zur aktiven Teilnahme auf dem Markt für personennahe Dienstleistungen zu bewegen (vgl. Spelsiek 2005).

## Incentivierung zur Beteiligung

### Barrieren der aktiven Beteiligung

Um die Bereitschaft der Nutzer zur aktiven Beteiligung zu fördern, sind in Anlehnung an Rimser (2014) allgemeine Voraussetzungen zu schaffen:

- Ermöglichen und Unterstützen: (Zeitliche) Ressourcen zur Nachbarschaftshilfe müssen geschaffen werden.
- Befähigen: Die potenziellen Helfer müssen in die Lage versetzt werden, unterstützende Dienstleistungen durchführen zu können.
- Motivieren: Intrinsische und extrinsische Anreize zur aktiven Beteiligung sind zu setzen.

Die Befähigung wird durch das Grundkonzept der INSELpro-App gewährleistet. Ein Matching-Algorithmus bewertet aufgrund der vorab eingegebenen persönlichen Kompetenzen und Fähigkeiten der potenziellen Helfer die eingegangene Suchanfrage und weist die geeignetsten Hilfswillgen aus, unter denen der Suchende sich „seinen“ Helfenden wählen kann. Seidel (2002) beschreibt dazu jeweils mehrere negativ wirkende Einflussfaktoren. Sie können sowohl durch entsprechendes Design des IT-Artefakts als auch durch begleitende Unterstützungsmaßnahmen sowie durch Incentivierungsmaßnahmen in ihrer Wirkung eingegrenzt oder überwunden werden.

### Motivatoren

**Abb. 3:**
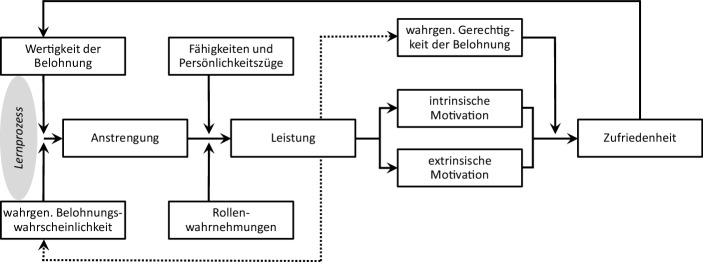
Lernprozess der individuellen Motivation. (Quelle: angepasst nach Porter und Lawler 1968)

Porter und Lawler (1968) beschreiben in ihrem Rückkopplungsmodell den Lernprozess von Individuen im Zusammenhang mit Anstrengung zur Leistung, folgender Belohnung und sich daraus einstellender Zufriedenheit. Wie Abb. 3 zeigt, spielen dabei neben persönlichen Faktoren (Fähigkeiten, Persönlichkeitszüge und Rollenwahrnehmungen) auch höchst individuelle Empfindungen über die Wahrscheinlichkeit des Eintreffens einer Erkenntlichkeit und ihrer Gerechtigkeit eine wichtige Rolle. Aus absolvierten Anstrengungs-Belohnungs-Zufriedenheits-Kreisläufen wird gelernt, welcher Aufwand in Zukunft für die gewünschte Belohnung und damit Zufriedenheit aufgewendet werden muss.

### Qualitätsmanagement

**Abb. 4:**
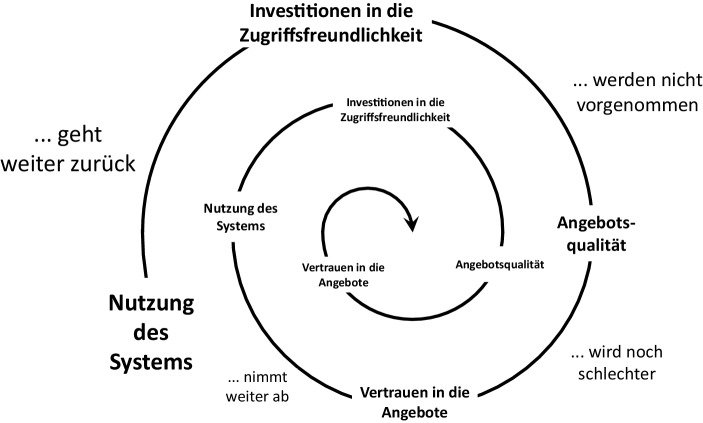
Todesspirale eines Wissensmanagement-Systems. (Quelle: angepasst nach Probst et al. 2012)

Die Qualität ist wichtiger Faktor zur Beteiligung von Freiwilligen. Eine eher niedrige Qualität von Plattform und Dienstleistungen führt dazu, dass sich immer weniger Nutzer zu Hilfsleistungen bereit erklären, woraufhin es für die Hilfesuchenden zunehmend schwieriger wird, noch Unterstützung zu erhalten. Die Gesamtqualität sinkt, was weitere Nutzer davon abhält, sich zu engagieren. Dieses Verhalten wird als „Todesspirale“ bezeichnet und ist in Abb. 4 skizziert.

Da die erbrachte Qualität einer Dienstleistung nicht sichergestellt werden kann, werden die Nutzer der Plattform zu Aussagen über die Nützlichkeit der erbrachten Hilfsleistung angeregt. Eine derartige individuelle Bewertung wird zu einem Durchschnittswert verdichtet und auf der Plattform angezeigt. Durch den Versandhändler Amazon und weitere „early adopters“ wie Hotelbuchungs- und -bewertungsportale ist ein 5‑Sterne-Bewertungssystem bekannt. Es bietet dem Kunden die Möglichkeit, seine Meinung zum Angebot kundzutun (beispielsweise zum gekauften Produkt oder besuchten Hotel), ohne den Aufwand auf sich nehmen zu müssen, eine Rezension als Text zu verfassen.

**Abb. 5:**
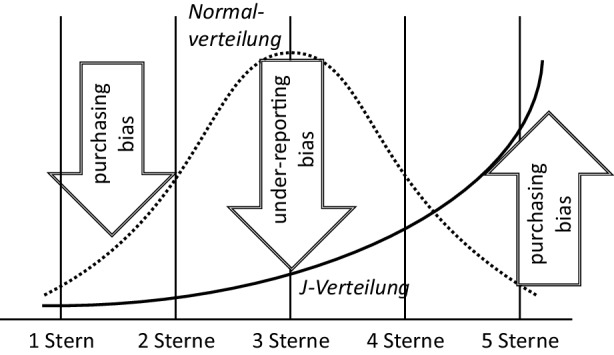
Abweichung bei der Verteilung von Bewertungen. (Quelle: eigene Darstellung in Anlehnung an Hu et al. 2009)

Produktbewertungen bei Online-Händlern folgen oftmals einer J‑Verteilung. Laut einer Untersuchung von Hu et al. (2009) erhalten aus den betrachteten Produktkategorien jeweils zwischen 70 und 80 % der Bewertungen im Analysezeitraum eine sehr gute Beurteilung mit vier oder fünf Sternen auf der Skala von 1–5. Die Gründe dafür liegen in zwei Selbstselektionseigenschaften der Käufer: Zum einen überwiegt die Zahl derjenigen, die das Produkt ohnehin kaufen wollen und sich bereits darüber informiert haben. Diese Käufer sind dann auch eher zufrieden und geben seltener eine negative oder neutrale Bewertung ab. Der „purchasing bias“ (Käufervoreingenommenheit) senkt somit die zu erwartende Zahl schlechter Bewertungen. Zum anderen werden Bewertungen häufiger von Käufern mit extrem positiven oder negativen Meinungen zum Produkt abgegeben, während die „lediglich“ zufriedenen, aber nicht euphorisierten Käufer gar keine Bewertung abgeben. Dieser Fall wird als „under-reporting bias“ (Untererfassung) bezeichnet (Hu et al. 2009). Eine schematische Darstellung dieser Verteilungsanomalie zeigt Abb. 5.

Ähnliches Verhalten ist auf einem Dienstleistungsmarkt anzunehmen. Auch hier sind relativ wenige neutrale Bewertungen zu erwarten, während die positiven Beurteilungen deutlich stärker vertreten sind. An dieser Stelle gilt daher ebenso die Regel, als Maßstab zur Bewertung nicht ausschließlich den Bewertungsdurchschnitt heranzuziehen, sondern mindestens auch die Standardabweichung. Für das Qualitätsmanagement besonders relevant sind Bewertungen unterhalb der Skalenmitte: Sie werden als erster Hinweis auf eine fragwürdige Güte der Hilfsleistung interpretiert, diese Hilfsangebote bedürfen erhöhter Aufmerksamkeit. Nutzer mit nennenswert vielen erhaltenen wie auch vergebenen Einträgen unter drei Sternen sind somit Qualitätsmanagern zu einer persönlichen Überprüfung vorzulegen.

## Incentivierung zur Beteiligung

Wie in Abschn. 3.1 erläutert, tragen Aufbau und Funktionalität der INSELpro-App an vielen Stellen zur Stärkung des Dienstleistungsmarktes bei, beziehungsweise helfen bei der Überwindung von Hürden. Zusätzlich relevante Faktoren sind die extrinsischen immateriellen und materiellen Belohnungen, die mit der aktiven Beteiligung an der einhergehen.

### Auszeichnungen

Auszeichnungen werden nach Frey und Neckermann (2006) bei der ökonomischen Betrachtung von Anreizsystemen oft vernachlässigt, finden aber zunehmend Aufmerksamkeit. Sie verfügen über eine Signalwirkung und motivieren damit andere Individuen intrinsisch, nach ihnen zu streben; gleichzeitig sind die Kosten äußerst gering.

Immaterielle Belohnungen der INSELpro-Plattform werden durch ein Statussymbol- und Auszeichnungssystem zuerkannt und tangieren damit die soziale Ebene der Anreizsetzung. Die Vergabe von Bonuspunkten kann automatisiert und abhängig von vordefinierten Bedingungen erfolgen, wie etwa der Zahl geleisteter Einsätze, der Reaktionszeit auf Anfragen oder auf Basis der Bewertungen durch die Dienstleistungsanfrager.

Implementiert ist zunächst ein Statussymbolkonzept (Badge), das aus der Summe geleisteter Hilfestellungen für jeden Nutzer abgeleitet wird (z. B. Symbole wie Kinderwagen, Fußabdruck, Fahrrad, Motorrad, Bus, Auto, Helikopter, Flugzeug, Rakete). Diese Status werden im Allgemeinen über die Mitgliedsdauer eines Nutzers auf der Plattform fortgeschrieben, repräsentieren die Beteiligung am Dienstleistungsaustausch und damit das Erfahrungslevel.

In einem zweiten Schritt wird die mit ihrer jeweiligen Bewertung gewichtete Anzahl der geleisteten Hilfseinsätze aufsummiert. Derjenige Nutzer mit der höchsten Quote wird am Monatsende als „Nachbarschaftsheld des Monats“ ausgezeichnet und bekommt ein entsprechendes Badge verliehen. Es wird wie die Statussymbole im persönlichen Profil und bei jedem Anfrage- oder Angebotseintrag des ausgezeichneten Nutzers angezeigt und dient damit als virtuelles Aushängeschild für besondere Verdienste um die Nachbarschaftshilfe.

### Qualitätsmanagement

Wie in Abschn. 3.3 erläutert, ist die Qualität der Angebote neben der zur Verfügung stehenden technischen Plattform ein wichtiger Aspekt für die Nutzung der Angebote im Rahmen der Vermittlung personennaher Dienstleistungen. Da eine INSELpro-vermittelte Leistungserbringung gewerbliche Dienstleister nicht ersetzen soll, durch den privaten Charakter einer Nachbarschaftshilfe keinen professionellen Anforderungen entsprechen muss sowie offline stattfindet, sind herkömmliche Ansätze des Qualitätsmanagements nicht zielführend.

Es gilt daher, die bei der Nutzung der Vermittlungsplattform anfallenden Daten zu operationalisieren, um die Vorschläge des Zuordnungsalgorithmus zu beeinflussen und einen lokalen Quartiersverantwortlichen als Qualitätsmanager zu unterstützen. Ihm/ihr werden diejenigen Nutzer bzw. abgeschlossenen Dienstleistungen zur manuellen Sichtung vorgeschlagen, die vom System als überprüfenswert klassifiziert werden.

### Bewertungen

Bewertungen von Produkten in Online-Shops sind meist J‑verteilt, weshalb die reine Betrachtung des Mittelwertes zur Einschätzung der Qualität nicht ausreichend ist (vgl. Abschn. 3.3). Dies ist auch für die Bewertung von Dienstleistungen zu erwarten. Für die INSELpro-Plattform werden daher nach Abschluss jeder Dienstleistung Bewertungen der Beteiligten über die Qualität der Dienstleistung und Zuverlässigkeit des Helfers bzw. Hilfesuchenden eingeholt, die aus Gründen der Bedienbarkeit lediglich über die Auswahl eines von jeweils drei Smileys (traurig, normal und lachend) charakterisiert werden.

Weichen die Bewertungen der Beteiligten stark voneinander ab (d. h. zufrieden vs. unzufrieden), wird dieser Fall an das Quartiersmanagement zur persönlichen Untersuchung gemeldet. Auch auffällig häufige unterschiedliche, gegenseitige Bewertungen oder kurzfristige Absagen werden angezeigt. Persönliche Rückfragen an die Beteiligten helfen dabei, Qualitäts‑, Kommunikations‑, Bedien- oder Zuordnungsprobleme zeitnah zu erkennen und an ihrer Lösung zu mitzuwirken.

Zusätzlich gehen die Bewertungen der Servicequalität in die Berechnung von geeigneten Helfern einer Suchanfrage ein. Besser bewertete Helfer werden bei gleicher Eignung aufgrund ihrer Kompetenzen prominenter angezeigt, was ebenfalls die Qualität der zu erbringenden Dienstleistung beeinflusst. Gleichzeitig steigt allerdings die Gefahr der Überlastung von sehr gut bewerteten Prosumern. Daher ist es wichtig, in der Hilfskultur zu verankern, dass die Absage einer Anfrage keinen negativen Akt darstellt. Die Bewertungen einzelner Nutzer werden wie auch die Auszeichnungen daher auf der Plattform lediglich im Rahmen einer Hilfsanfrage in der Liste potenzieller Unterstützer angezeigt. Eine Art genereller Nutzerübersicht oder Mitgliederliste ist weder öffentlich noch für registrierte Prosumer einsehbar.

Der Einfluss älterer Bewertungen kann mit fortschreitender Zeit verringert werden, um sich den eventuell geänderten Lebens- und Hilfsumständen anzupassen. So ist es wenig sinnvoll, einen ehemals sehr guten Anbieter auch dann noch auf vorderen Plätzen der Vorschlagsliste anzuzeigen, wenn dieser aufgrund einer veränderten Lebenssituation (z. B. nun selber ein Pflegefall) zu Hilfsangeboten gar nicht mehr fähig ist. Zunächst ist vorgesehen, weiter als ein Jahr zurückliegende Bewertungen nur noch zu 50 % im Matching-Algorithmus zu berücksichtigen. Die Werte können durch das Quartiersmanagement den realen Nutzungsbedingungen angepasst werden.

### Weitere Motivatoren

Neben allgemeinen, intrinsischen Motivationsaspekten wie der Freude am Helfen und dem Erleben der Nützlichkeit einer Hilfsdienstleistung, werden auch weitere Motivatoren eingeführt:

Regelmäßige, öffentlich angekündigte Vernetzungstreffen für die lokalen Anwohner dienen der Steigerung der Projektbekanntheit und der App vor Ort. Hier kommen potenzielle Neu-Prosumenten mit bereits aktiven Hilfegebern- und Empfängern zusammen, welche die „Neuen“ mit in das soziale Netzwerk der Nachbarschaft integrieren und als Projektbeteiligte binden. Das Vorstellen von „Erfolgsgeschichten“ der nachbarschaftlichen Dienstleistungen beispielsweise im Rahmen der persönlichen Auszeichnung besonders verdienter Prosumenten aus Abschn. 4.1 kann ebenfalls dabei helfen, die Beteiligung am Netzwerk zu steigern bzw. hoch zu halten. Darauf zielt auch die Verlosung von durch örtliche Unternehmen gesponserten Kleinstpreisen an aktive Nutzer auf Geber- und Nehmerseite über die App ab.

## Zusammenfassung und Ausblick

Anders als beispielweise Facebook mit seinen Ortsgruppen bietet das Projekt INSELpro den neuartigen Ansatz, zur Vermittlung von Hilfesuchenden und Hilfswilligen mithilfe digitaler Medien die Auswahl potenzieller Unterstützer durch eine Zuordnungsmatrix aus den für die jeweilige Anfrage benötigten und bei Nutzern vorhandenen Fähigkeiten einzuschränken. Hierdurch wird ermöglicht, potenzielle Helfer gezielt persönlich anzusprechen. Die vorgestellten Maßnahmen zur Incentivierung tragen dazu bei, die Motivation zur Beteiligung an der Nachbarschaftshilfe inner- wie außerhalb der Plattform zu stimulieren, um die für diese Art der Vermittlung von Dienstleistungen notwendige Anzahl tatsächlich aktiver User zu erreichen bzw. beizubehalten. Sie nutzen die vorhandenen Möglichkeiten und Daten innerhalb der Plattform, um insbesondere die Hilfswilligen zu stimulieren, sie gleichzeitig aber auch nicht durch zu umfangreiche Abfragen zu demotivieren (Abfrage lediglich von Zuverlässigkeit des Helfenden und Qualität der Hilfsleistung). Gleichzeitig bieten sie die Möglichkeit, Offline-Maßnahmen wie die Auszeichnung besonders hilfsbereiter Nachbarn vorzunehmen.

Nach den ersten Tests von Zuordnungsalgorithmen und App auf Basis der dem Projekt zugrundeliegenden Personas beginnt im Frühjahr 2020 zunächst ein Usability-Test, der auch bereits die Abbildung der im Beitrag genannten Onlinefunktionen für Incentivierung und Qualitätsmanagement berücksichtigt. Nach einer Überarbeitung des Systems basierend auf den Rückmeldungen der Usability-Tester werden ab dem dritten Quartal die ersten Bewohner des bis dahin fertiggestellten Quartier Langseestraße in Nürnberg-Mögeldorf das System benutzen. Ziel ist es, die Nutzung der INSELpro-Plattform bis August 2021 auf den ganzen Stadtteil Mögeldorf auszuweiten und damit eine aktive und lebendige Community der Nachbarschaftshilfe zu begründen.

Die Evaluation der Wirksamkeit der Incentivierungsmaßnahmen ist in Arbeit und wird gemeinsam mit der finalen Umsetzung in einem späteren Beitrag vorgestellt.

## Footnotes

1 Akronym steht für „*In*telligentes *Se*rvicesystem für *l*okal vernetzte *Pro*sumenten“; siehe auch www.inselpro.de.

2 Quartier Langseestraße mit Service-Wohnen, siehe auch www.t1p.de/langseestrasse und www.diakonie-moegeldorf.de/leistungen/alter/.
